# 14-3-3 Dysfunction in Dorsal Hippocampus CA1 (dCA1) Induces Psychomotor Behavior *via* a dCA1-Lateral Septum-Ventral Tegmental Area Pathway

**DOI:** 10.3389/fnmol.2022.817227

**Published:** 2022-02-14

**Authors:** Jiajing Zhang, Meaghan Navarrete, Yuying Wu, Yi Zhou

**Affiliations:** Department of Biomedical Sciences, Florida State University College of Medicine, Tallahassee, FL, United States

**Keywords:** 14-3-3, dorsal hippocampus CA1, hippocampal hyperactivity, lateral septum (LS), ventral tegmental area (VTA), psychomotor behavior, chemogenetics, dopamine

## Abstract

While hippocampal hyperactivity is implicated in psychosis by both human and animal studies, whether it induces a hyperdopaminergic state and the underlying neural circuitry remains elusive. Previous studies established that region-specific inhibition of 14-3-3 proteins in the dorsal hippocampus CA1 (dCA1) induces schizophrenia-like behaviors in mice, including a novelty-induced locomotor hyperactivity. In this study, we showed that 14-3-3 dysfunction in the dCA1 over-activates ventral tegmental area (VTA) dopaminergic neurons, and such over-activation is necessary for eliciting psychomotor behavior in mice. We demonstrated that such hippocampal dysregulation of the VTA during psychomotor behavior is dependent on an over-activation of the lateral septum (LS), given that inhibition of the LS attenuates over-activation of dopaminergic neurons and psychomotor behavior induced by 14-3-3 inhibition in the dCA1. Moreover, 14-3-3 inhibition-induced neuronal activations within the dCA1-LS-VTA pathway and psychomotor behavior can be reproduced by direct chemogenetic activation of LS-projecting dCA1 neurons. Collectively, these results suggest that 14-3-3 dysfunction in the dCA1 results in hippocampal hyperactivation which leads to psychomotor behavior *via* a dCA1-LS-VTA pathway.

## Introduction

The heterogeneity of psychiatric disorders poses major difficulties in deciphering their underlying etiology. Thus, unraveling the circuitry basis of psychiatric symptoms holds promise for identifying novel circuit-level targets for future treatment development. It is well recognized that the mesolimbic dopamine (DA) system, which originates from DA neurons in the ventral tegmental area (VTA), is critical for salience attribution, which is impaired in patients experiencing psychosis (Laruelle et al., 1999; Abi-Dargham et al., 2009; Howes and Nour, 2016). As the DA system is tightly modulated by excitatory and inhibitory inputs from the cortical and subcortical regions in response to both internal and external stimuli, a pathological DAergic hyperactivation is thought to be secondary to dysfunction in susceptible brain regions that regulate the VTA (Lodge and Grace, 2011; Howes et al., 2015; Howes and Nour, 2016). In particular, cortical and hippocampal anomalies are highly implicated in psychiatric disorders, which may explain why the brain’s failures in processing sensory information could give rise to psychosis.

Alterations in structure and physiology of the hippocampus (HP) have been consistently linked to schizophrenia (Medoff et al., 2001; Schobel et al., 2009a; Talati et al., 2014; McHugo et al., 2019). Specifically, human imaging studies identified hippocampal hyperactivity associated with psychosis (Talati et al., 2014; Dugré et al., 2019; McHugo et al., 2019). Consistent with this, studies using transgenic or developmental rodent models of schizophrenia associate a loss of hippocampal GABAergic interneurons activity (Belforte et al., 2010; Gilani et al., 2014) or increased hippocampal neuronal activity (higher firing rate, increased c-Fos, etc.) with psychomotor behavior (Lodge and Grace, 2007; Procaccini et al., 2011). Additionally, such behavioral abnormality can be rescued by restoring hippocampal GABAergic interneurons (Marissal et al., 2018) or by pharmacological or chemogenetic inhibition of hippocampal excitatory neurons (Maksimovic et al., 2014; Aitta-Aho et al., 2019). Collectively, these findings support a hypothesis in which psychosis is a consequence of hippocampal excitation/inhibition (E/I) imbalance.

In individual neurons, synaptic inputs are highly regulated to ensure proper excitatory or inhibitory activity across dendritic segments (Gao and Penzes, 2015). Numerous genes derived from linkage and association studies of psychiatric disorders encode proteins involved with synaptic processes (Kirov et al., 2012; Fromer et al., 2014). Among them are several genes that encode members of the 14-3-3 family of proteins which are particularly enriched at synapses (Bell et al., 2000; Jia et al., 2004; Middleton et al., 2005; Wong et al., 2005; Ikeda et al., 2008). Accumulating evidence reveals that 14-3-3 proteins are important modulators of synaptic transmission and plasticity (Zhang and Zhou, 2018), thus making them potential therapeutic targets. Transgenic mouse models with 14-3-3 deficiency exhibit synaptic, network activity, and behavioral alterations that correspond to core schizophrenia phenotypes (Ramshaw et al., 2013; Qiao et al., 2014; Foote et al., 2015; Jaehne et al., 2015; Xu et al., 2015; Jones et al., 2021). Most interestingly, recent work reveals that adeno-associated virus (AAV) mediated 14-3-3 isoform-independent inhibition specifically in the dHP CA1 (dCA1) pyramidal neurons induces psychomotor behavior in wild type (WT) mice provoked by novel environment (Graham et al., 2019). However, the circuitry mechanism underlying such hippocampal dysfunction-induced psychomotor behavior and how 14-3-3 deficiency in the dCA1 alters hippocampal E/I balance and disturbs downstream neural activities remain to be determined.

In this study, we demonstrate that 14-3-3 dysfunction in the dCA1 induces psychomotor behavior in mice *via* over-activation of the lateral septum (LS) neurons, which results in increased VTA DA neuronal activity. We also reveal that 14-3-3 inhibition-induced alterations in neuronal activity and behavior are likely due to an increased dCA1 neuronal activation, and provided evidence showing that direct chemogenetic activation of the dCA1-LS pathway is sufficient to induce locomotor hyperactivity in mice. Together, these findings demonstrate how the loss of function of one pivotal family of proteins in the hippocampus may contribute to a shift in E/I balance and induces psychotic-like behavioral abnormality in mice through dysregulation of the downstream pathway.

## Materials and Methods

### Experimental Animals

Both male and female adult (3–6 months old) wildtype (C57BL/6J), DAT-cre [B6.SJL-Slc6a3tm1.1(cre)Bkmn/J, Jackson Laboratory], and CaMKIIa-cre [B6.Cg-Tg(Camk2a-cre)T29-1Stl/J, Jackson Laboratory] mice were included in this study. Mice were housed on a 12 h light/dark cycle and provided ad libitum access to water and food. Prior to stereotaxic surgical injection, mice were housed in groups (2–4 per cage). Littermates were randomly assigned to experimental groups or control groups. Following the surgical procedure, mice were subsequently single caged until all experimental procedures were finished. Mice were handled daily for 2 weeks prior to behavioral tests, which were conducted during the light cycle. All experiments were carried out in accordance with Florida State University’s laboratory animal care and use guidelines and approved by the Florida State University Animal Care and Use Committee.

### Viral Vectors

The AAV2/9-CaMKIIa-YFP-difopein (titer at 7.82 × 10^12^ v.g./ml), AAV2-CaMKIIa-tdTomato (titer at 4.42 × 10^12^ v.g./ml), and AAV2/9-CMV-DIO-EGFP (titer at 9.05 × 10^12^ v.g./ml) were constructed and produced by OBiO Technology (Shanghai) Corp., Ltd. Briefly, cDNA encoding YFP-difopein, tdTomato, or DIO-EGFP was subcloned into a rAAV vector. The viral vectors were then produced using the triple transfection method in HEK 293 cells and AAV titers were determined by real-time PCR. Control vector (AAV2-CaMKIIa-YFP, titer at 5.1 × 10^12^ v.g./ml) was purchased from the UNC viral core facility. For chemogenetic manipulations, AAV5-hSyn-hM4D(Gi)-mCherry (Addgene #50475, titer at 1.2 × 10^13^ v.g./ml), AAV5-hSyn-DIO-hM4D(Gi)-mCherry (Addgene #44362, titer at 8 × 10^12^ v.g./ml), AAV5-hSyn-mCherry (Addgene #114472, titer at 2.8 × 10^13^ v.g./ml), AAV5-hSyn-hM3D(Gq)-mCherry (Addgene #50474, titer at 1 × 10^13^ v.g./ml), AAV5-hSyn-DIO-hM3D(Gq)-mCherry (Addgene #44361, titer at 1 × 10^13^ v.g./ml), and AAVretro-hSyn-Cre-WPRE-hGH (Addgene #105553, titer at 2.1 × 10^13^ v.g./ml) were purchased from Addgene. Upon arrival, all viral vectors were aliquoted and stored at −80°C prior to stereotaxic injections.

### Stereotaxic Viral Injections

Mice were anesthetized with intraperitoneal (i.p.) injections of a mixture of ketamine (100 mg/kg)/xylazine (10 mg/kg) and placed in a stereotaxic frame (David Kopf Instruments, Tujunga, CA). The animal’s skull was exposed *via* a small incision on the scalp. Small burr holes were made directly above the viral injection sites unilaterally or bilaterally using a micro-precision drill. For micro-injections, Hamilton syringes (10 μl, 33-gauge) loaded with AAV virus or tracer were slowly lowered into the target area according to the corresponding coordinates: dCA1 (AP: − 2.0 mm, ML: ± 1.5 mm, DV: − 1.1 mm from dura); LS (AP: + 0.6 mm, ML: ± 0.7 mm, DV: − 2.25 mm from dura with a 10° coronal rotation angle); VTA (AP: − 3.0 mm, ML: ± 0.5 mm, DV: − 4.25 mm from dura); NAc (AP: + 1.3 mm, ML: ± 0.8 mm, DV: − 4.3 mm from dura); MM (AP: − 2.9 mm, ML: ± 0.5 mm, DV: − 4.9 mm from dura). Virus or tracer (0.5–1 ul) was slowly injected at 75 nl/min. Injection needles were left in place for an additional 10 min to assure adequate viral delivery before slowly being withdrawn. The scalp incision was then closed and treated with topical neomycin. For postoperative care, ketoprofen (5 mg/kg in 0.05 ml saline) was used for pain relief immediately following surgery. Mice were allowed 2 weeks for expression of viral proteins and recovery from surgery before beginning behavioral testing.

### Pharmacology

The stock solution of clozapine (1 mg/ml, TOCRIS) was prepared in 0.1 N HCl and diluted in saline. On the day of the experiment, 2 mg/kg clozapine in 0.2 ml saline was prepared for injections. Compound 21 dihydrochloride (C21) was purchased from Hello Bio. For hM4D(Gi)-mediated inhibition and hM3D(Gq)-mediated excitation, 1–2 mg/kg C21 in 0.2 ml saline was used. Diluted pharmacological agents (or saline as control) were intraperitoneally (i.p.) given to each testing subject 30 min prior to open field testing.

### Open Field Testing

All mice were habituated in the behavioral room for a minimum of 30 min prior to beginning experimental sessions. To assess locomotive behavior, mice were placed into a square open field arena (Med Associates Open Field Arena, 43.2 cm × 43.2 cm × 30.5 cm, with IR photo-beam sensors) and the total distance traveled in 30 min was measured using Med Associates Activity Monitor software.

### Immunofluorescence and Imaging

Mice were anesthetized with ketamine/xylazine and perfused with 0.1 M phosphate-buffered saline (PBS), followed by 4% paraformaldehyde (PFA) in 0.1 M PBS. Brains were extracted and post-fixed overnight at 4°C in 4% PFA before transferring to PBS solution. A vibratome (Leica Microsystems) was used for brain sectioning at 40 μm. Brain slices were collected and stored in PBS with 0.1% sodium azide. For immunohistochemistry, brain sections were blocked in a PBS solution (PBST) containing 10% goat serum and 0.7% Triton-X for 1 h at room temperature. The brain sections were then incubated overnight (two nights for c-Fos) at 4°C with primary antibodies. Then after three PBST washes, the brain sections were incubated with secondary antibodies at room temperature for 2 h (4 h for c-Fos). The sections were rinsed three times with PBST, followed by one PBS wash. If DAPI counterstaining was needed, brain sections were then incubated in PBS with 300 nM DAPI (Invitrogen, P3571) for 10 min before being mounted using an antifade mounting medium (VECTASHIELD). Keyence and Zeiss confocal microscopes were used for imaging.

Primary antibodies including rabbit anti-c-Fos (abcam, ab190289); mouse anti-TH (Millipore, MAB318); rabbit anti-Alexa Fluor 488 IgG (Invitrogen, Cat#A11094); mouse anti-GAD67 (Millipore, MAB5406); rabbit anti-GABA (Sigma-Aldrich, A2052); mouse anti-beta subunit Cholera Toxin (abcam, ab62429); mouse anti-cre recombinase (Millipore, MAB3120); and secondary antibodies including Alexa Fluor 647 donkey anti-rabbit IgG(H + L; Invitrogen, A31573); mouse IgG F(c) Antibody DyLight^TM^ 405 Conjugated (Rockland, Cat# 610-146-003); anti-rabbit IgG(H + L) FITC (SouthernBiotech, 4050-02); and Alexa Fluor 647 donkey anti-mouse IgG(H + L; Invitrogen, A31571) were used in this study.

### Anterograde and Retrograde Tracing

For dual anterograde and retrograde tracing, 0.8 ul recombinant cholera toxin-b conjugated to Alexa Fluor 488 (CTB488, Invitrogen Cat#C34775) in PBS was unilaterally injected into the VTA of WT mice, followed by 0.5 ul AAV2-CaMKIIa-tdTomato (OBiO Technology) injected into the dCA1 ipsilateral to the CTB-injected VTA. For retrograde tracing from the LS, 0.5 ul CTB488 was unilaterally injected into the targeted LS region. Mice (*n* = 4 each tracing experiment) were perfused on day 14 following the surgery. Coronal brain slices (40 μm) were sectioned and immunohistochemistry against Alexa Fluor 488 IgG was performed to enhance CTB signals. LS sections containing CTB-labeled VTA-projecting cells were further co-stained with anti-GAD67 or anti-GABA for identification of cell type. Tracing results were examined under a Zeiss confocal microscope with 10×, 20×, and 60× objectives.

### c-Fos Induction and Quantifications

To minimize unrelated c-Fos expression, all virally transduced mice were habituated in the behavioral room for at least 4 h prior to testing. For experiments aiming to assess c-Fos expression associated with difopein-induced locomotive hyperactivity, four groups of mice were used: difopein-OFT, YFP-OFT, difopein-handled only, and YFP-handled only. After habituation, difopein-OFT mice and YFP-OFT mice were put through 30-min OFT, returned to home cages, and then perfused 60 min following the end of OFT. Difopein- and YFP-handled only mice were briefly handled and perfused 90 min after returning to their home cages. Each mouse brain was sectioned into six sets of 40 μm coronal slices. For whole brain c-Fos analysis, one set of slices from each brain was used for immunohistochemistry against c-Fos (Alexa Fluor 647 donkey anti-mouse IgG(H + L) was used as a secondary antibody). A Keyence microscope (20× objective) was used to collect images for brain-wide c-Fos quantification in 28 brain nuclei selected based on three criteria: (1) c-Fos expression in the region was previously found to be associated with open field exposure (Badiani et al., 1998; Hale et al., 2008); (2) the region showed c-Fos activity in any of the four groups following OFT or handling; (3) the region was identified as having efferent projections to the VTA and/or receive input from the dHP (Strange et al., 2014; Beier et al., 2015). The regions selected were: nucleus accumbens core (NAcore), nucleus accumbens shell (lateral part, NAsh L), nucleus accumbens shell (medial part, NAsh M), cingulate cortex (Cg), secondary motor cortex (M2), primary motor cortex (M1), agranular insular cortex (AI), claustrum (Cl), dorsal endopiriform nucleus (DEn), piriform cortex (Pir), lateral septum (rostral part, LSr), lateral septum (caudal, LSc), bed nucleus of the stria terminalis (BST), paraventricular thalamic nucleus (PVT), central amygdaloid nucleus (Ce), lateral amygdaloid nucleus (LA), basolateral amygdaloid nucleus (BLA), medial amygdaloid nucleus (Me), basomedial amygdaloid nucleus (BMA), lateral hypothalamus (LH), reticular thalamic nucleus (RT), dHP CA1 (dCA1), dHP CA3 (dCA3), dentate gyrus (DG), lateral entorhinal cortex (LEnt), ventral hippocampus CA1 (vCA1), subiculum (Sub), and mammillary body (MM). The number of c-Fos-ir cells in each image was programmatically counted using ImageJ.

For VTA cell-type specific c-Fos analysis, VTA-containing coronal sections from another set of slices were used. Brain slices were co-stained with primary antibodies against c-Fos and tyrosine hydroxylase, followed by secondary antibodies Alexa Fluor 647 donkey anti-mouse IgG(H + L) and 405 conjugated anti-mouse IgG. Fluorescent images were collected using a Zeiss confocal microscope. All images were taken under the same settings and processed identically to avoid artificial representations of data. Z-stack images of the VTA from four slices (200 μm apart, correspond roughly to Bregma − 3.1 mm, − 3.3 mm, − 3.5 mm, and − 3.7 mm in the mouse brain atlas) per animal were collected using the 20× objective, and neurons expressing c-Fos and/or TH were manually counted. TH-ir cells in the VTA were considered c-Fos-positive if the c-Fos signal clearly overlapped with the neuronal soma of the TH-ir cells. The sum of cells counted from four sections on one hemisphere of each mouse was used for statistical analysis.

For the experiment analyzing c-Fos expression in VTA-projecting LS cells, virally transduced mice were put through 30-min OFT 14 days after the surgery and then perfused 60 min following the end of the test. LS-containing sections from one set of slices were used for immunohistochemistry co-staining of c-Fos and CTB. Alexa Fluor 647 donkey anti-mouse IgG(H + L) and 405 conjugated anti-mouse IgG were used as secondary antibodies. Z-stack images of the LS from six slices (200 μm apart) per mouse were collected using Zeiss confocal with a 10× objective. Neurons expressing c-Fos and/or CTB were manually counted. CTB-ir cells in the LS were considered c-Fos-positive if the c-Fos signal clearly overlapped with neuronal soma of the CTB-ir cells. The total cell numbers counted from six sections on one hemisphere per mouse were used for statistical analysis.

### Chemogenetic Manipulations

To validate the effect of C21-mediated chemogenetic activation or inhibition, saline or C21 was administered to hM3D- or hM4D-injected mice 30 min prior to OFT. Animals were then perfused 60 min after 30 min OFT. Brain sections containing hM3D or hM4D injection sites from one set of slices per brain were used for immunohistochemistry against c-Fos. Z-stack images were taken using a Zeiss confocal microscope with a 20× objective. Neurons expressing hM3D or hM4D were considered c-Fos-positive if the c-Fos signal clearly overlapped with neuronal soma of the mCherry-positive cells. The number of cells expressing mCherry and/or c-Fos were manually counted.

For experiments using hM4D, one set of virally transduced mice from each group was first put through 30-min OFT with i.p. injections of saline 30 min prior to the first trial. Two weeks later, these mice were given i.p. injections of C21 prior to the second trial. Mice were perfused 60 min after the end of the second trial for c-Fos analysis. To assess the effect of repeat testing, a separate set of virally transduced mice was given C21 administration prior to the first trial and was given saline injections prior to the second trial.

### Statistical Analysis

Power analysis was performed to ensure the appropriately minimal number of mice is used in each experimental context. Our considerations are based on a significant level (alpha set at 0.05), power set at 80%, and effective size which was established through our published and preliminary studies. Following *post-hoc* histological confirmation of viral infections, only mice with accurate viral infections were included in data analysis. All data were analyzed using Prism 7.01 (GraphPad software). Statistical analyses were performed using two-tailed unpaired Student’s t-tests, paired Student’s t-tests, or two-way ANOVA when appropriate. When homogeneity of variance was violated, a Welch test was used as a correction. Values are reported as mean ± standard error of the mean (SEM). The cutoff value of significance was *P* = 0.05. Symbols used: *p < 0.05; **p < 0.01; ***p < 0.001; ns, not significant.

## Results

### Locomotor Hyperactivity Induced by 14-3-3 Dysfunction in the dCA1 Is Attenuated by Clozapine Administration

**Figure 1:**
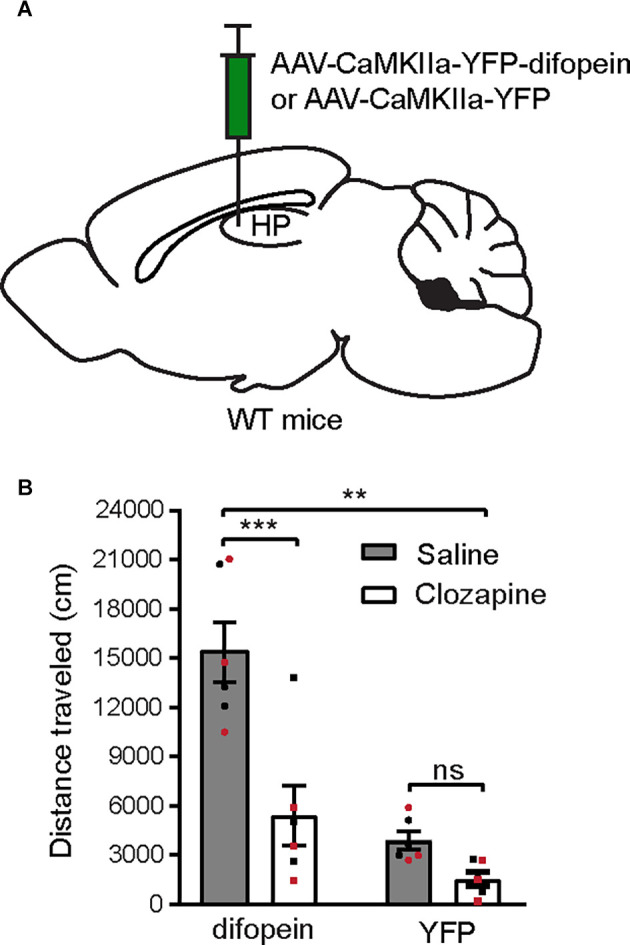
Systemic clozapine administration attenuates locomotor hyperactivity induced by 14-3-3 dysfunction in the dCA1. **(A)** Schematic depiction illustrating viral transduction of the dHP CA1 of WT mice with AAV-CaMKIIa-YFP-difopein or AAV-CaMKIIa-YFP. **(B)** Novelty-induced locomotor activity (distance traveled) of difopein- or YFP-injected mice during OFT following saline or clozapine (2 mg/kg) administration [*n* = 6 (3 males and 3 females)/group; two-way ANOVA, Drug × Virus interaction *F*_(1,20)_ = 8.214, **p = 0.0096, *post hoc* multiple Student’s unpaired two-tailed t-tests; difopein, ***p < 0.0001; YFP, *p* = 0.2239]. All data are presented as mean ± SEM. Data points from male and female mice are shown as black and red symbols, respectively. ns, not significant.

Novelty-induced locomotor hyperactivity in rodents are thought to share the underlying neurobiology of psychosis due to their similar pharmacological responses to antipsychotics and psychostimulants (van den Buuse, 2010). Therefore, novel open field exposure is commonly used to evaluate psychosis-like behavior in rodents. We previously demonstrated that AAV-mediated 14-3-3 inhibition in the dCA1 alone induces locomotor hyperactivity (Graham et al., 2019). Here, we examined the effect of clozapine, a DA receptor-targeting antipsychotic drug, on attenuating this behavioral abnormality. 14-3-3 inhibition in the dCA1 subregion was achieved by utilizing a previously established AAV to drive regional expression of a YFP-fused isoform-independent dimeric fourteen-three-three peptide inhibitor (difopein) under CaMKIIa promotor (AAV-difopein; Figure 1A; Masters and Fu, 2001). Consistent with previous findings, the difopein-injected WT mice exhibited significantly increased novelty-induced locomotor activity during 30-min open field testing (OFT; Figure 1B). Interestingly, administration of clozapine at a non-sedative dose (2 mg/kg) was sufficient to normalize the locomotor activity of the difopein-injected mice (Figure 1B). Given the preferential, although not selective, effect of clozapine on antagonizing DA transmission (Kapur and Seeman, 2001), these results suggest that a hyperactive DA system may underlie the locomotor hyperactivity induced by 14-3-3 inhibition in the dCA1.

### 14-3-3 Dysfunction in dCA1 Triggers Robust C-Fos Expression in VTA DA Neurons During OFT

**Figure 2:**
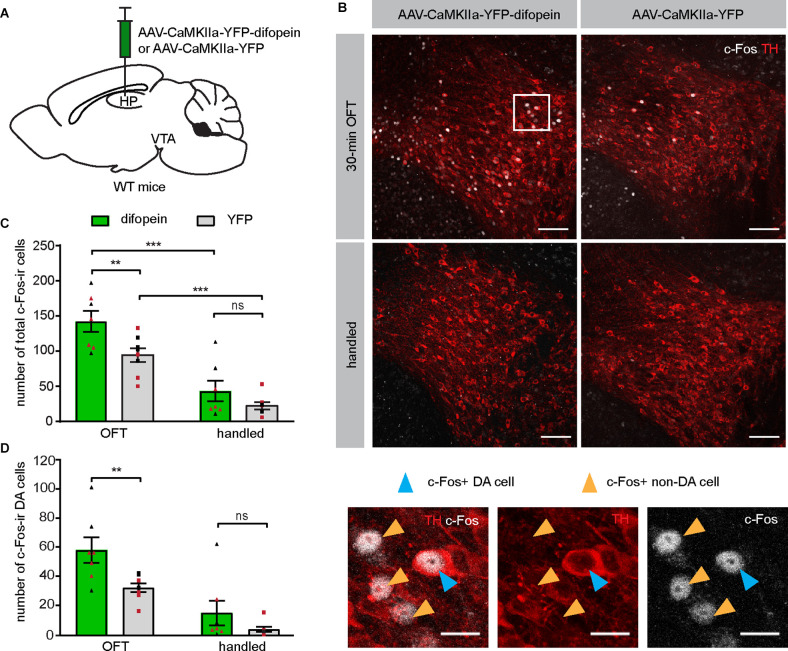
Inhibition of 14-3-3 in the dCA1 increases the number of c-Fos-ir cells in the VTA during OFT. **(A)** Schematic depiction illustrating viral transduction of the dHP CA1 of WT mice with AAV-CaMKIIa-YFP-difopein or AAV-CaMKIIa-YFP. **(B)** Top, representative confocal images of c-Fos and TH co-staining revealing activation of neurons in the VTA of difopein- or YFP-injected mice following either 30-min OFT or gentle handling. Scale bar = 100 μm. Bottom, magnified representative image. Blue arrowheads point to examples of c-Fos and TH co-labeled cells (putative c-Fos-ir DA neurons). Orange arrowheads point to examples of c-Fos-ir only cells (putative c-Fos-ir non-DA neurons). Scale bar = 20 μm. **(C)** Total numbers of c-Fos-ir cells in the VTA of difopein- [*n* = 7 (3 males and 4 females)/group] or YFP-injected mice [*n* = 8 (4 males and 4 females)/group] following OFT or handling (multiple Student’s unpaired two-tailed t-tests; difopein-OFT vs. YFP-OFT, **p = 0.0059; difopein-handled vs. YFP-handled, *p* = 0.2070; difopein-OFT vs. difopein-handled, ***p < 0.001; YFP-OFT vs. YFP-handled, ***p < 0.001). **(D)** Numbers of c-Fos and TH co-labeled cells (putative c-Fos-ir DA neurons) in the VTA of difopein- or YFP-injected mice following OFT or handling (multiple Student’s unpaired two-tailed t-tests; OFT, **p = 0.0049; handled, *p* = 0.1968). All data are presented as mean ± SEM. Data points from male and female mice are shown as black and red symbols, respectively. ns, not significant.

Having established that difopein-induced locomotor hyperactivity is sensitive to clozapine administration, we then sought to directly evaluate whether DA neuronal activity was altered during such behavior. Specifically, we focused on DAergic activity in the VTA, given that a hyperactive mesolimbic DA pathway is thought to give rise to psychosis (Boekhoudt et al., 2016). We injected AAV-difopein or AAV-YFP into the dCA1 of WT mice (Figure 2A) and examined the expression of c-Fos protein in the VTA following OFT. This protocol induced significantly increased numbers of total c-Fos-immunoreactive (ir) cells in the VTA of both difopein- and YFP-injected mice compared with their handled-only controls (Figures 2B,C), which is consistent with a previous report (Bourgeois et al., 2012) and suggests that the OFT induced neuronal activation in the VTA. Furthermore, among the mice that were exposed to the open field arena, we found a significantly higher total number of c-Fos-ir cells in the VTA of difopein-injected mice (Figure 2C). This indicates that 14-3-3 inhibition in the dCA1 further increases VTA neuronal activation in OFT.

It is known that the VTA comprises DAergic, GABAergic, glutamatergic, and co-releasing neurons (Yoo et al., 2016; Kim et al., 2019). To specifically identify the activation of DA neurons in virus-injected mice, we performed dual immunohistochemistry against tyrosine hydroxylase (TH) and c-Fos on VTA-containing brain sections. A significantly higher number of double-labeled neurons was found in the VTA of difopein-injected mice compared to control, indicating increased activation of DA neurons induced by 14-3-3 inhibition in the dCA1 during OFT (Figure 2D). Interestingly, we did not observe any statistically significant differences in either total or TH co-labeled c-Fos-ir cell numbers between difopein- and YFP-injected groups that were handled only (Figures 2C,D). This might suggest that inhibition of 14-3-3 disrupts the function of dCA1 in processing novel environmental stimuli and thus results in over-activation of the VTA DA neurons during OFT.

### Chemogenetic Inhibition of VTA DA Neurons Attenuates Difopein-Induced Locomotor Hyperactivity

**Figure 3:**
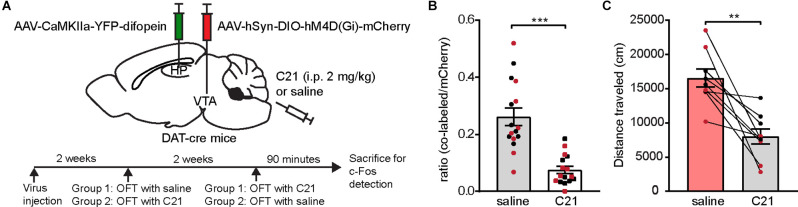
Chemogenetic inhibition of VTA DA neurons attenuates locomotor hyperactivity induced by 14-3-3 inhibition in the dCA1. **(A)** Schematic depiction illustrating (top) DAT-cre mice transduced with AAV-CaMKIIa-YFP-difopein in the dHP CA1 and cre-dependent hM4D in the VTA, and (bottom) experimental timeline. **(B)** Ratio of c-Fos/hM4D co-labeled DA cells to total hM4D-expressing DA cells in the VTA following OFT with saline or C21 administration (*n* = 16 sections from 2 male and 2 female mice/treatment, Student’s unpaired two-tailed t-test with Welch’s correction, ***p < 0.0001), indicating an effective reduction of VTA DA neuronal activity using chemogenetic manipulation. **(C)** Distance traveled for virally transduced DAT-cre mice during OFT following either saline or C21 administration [*n* = 9 (4 males and 5 females), Student’s paired two-tailed t-test, **p = 0.0021]. All data are presented as mean ± SEM. Data points from male and female mice are shown as black and red symbols, respectively.

Next, we directly tested whether over-activation of VTA DA neurons is necessary for 14-3-3 dysfunction-induced locomotor hyperactivity. We employed a chemogenetic DREADD (Designer Receptors Exclusively Activated by Designer Drugs) approach, which allows us to regulate neuronal activity with spatiotemporal precision. To selectively manipulate DA neurons, we injected an AAV expressing a Cre-recombinase-dependent inhibitory DREADD (AAV-hSyn-DIO-hM4D(Gi)-mCherry) bilaterally into the VTA of DAT-cre mice in addition to bilateral AAV-difopein injections into the dCA1 (Figure 3A). To circumvent potential off-target effects of clozapine-N-oxide on locomotion, we used an alternative DREADD agonist, Compound 21 (C21), for acute activation of designer receptors in this study (Manvich et al., 2018; Thompson et al., 2018). C21 administration (2 mg/kg) significantly reduced the number of c-Fos-expressing hM4D-positive neurons (Figure 3B), demonstrating an effective C21-mediated inactivation of DA neurons. Interestingly, we found that C21-mediated inhibition of VTA DA neurons is sufficient in attenuating locomotor hyperactivity in difopein-injected mice (Figure 3C), suggesting that DA activation is necessary for difopein-induced locomotor hyperactivity. Together, these findings demonstrate that activation of VTA DA neurons plays a significant role in the locomotor hyperactivity induced by 14-3-3 inhibition in the dCA1.

### 14-3-3 Inhibition in the dCA1 Induces Robust C-Fos Expression in the LS, a Relay Between the dCA1 and VTA

**Figure 4:**
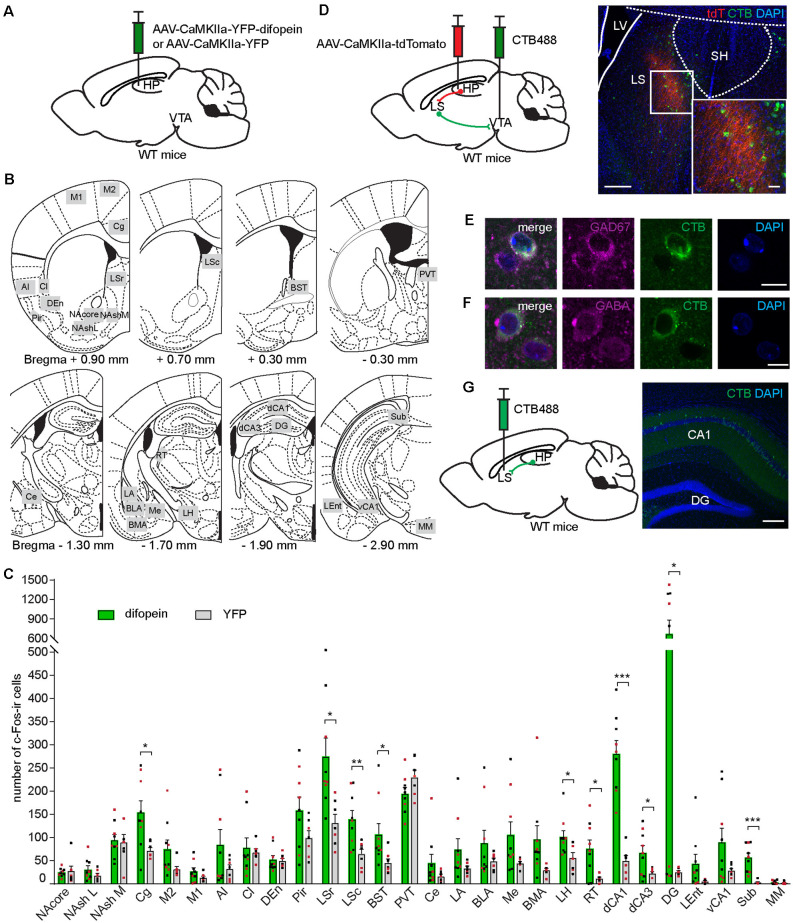
Inhibition of 14-3-3 in the dCA1 increases c-Fos expression in the LS, an anatomical relay between the dCA1 and VTA. **(A)** Schematic depiction illustrating viral transduction of WT mice dHP CA1 with AAV-CaMKIIa-YFP-difopein or AAV-CaMKIIa-YFP. **(B)** Line drawings (adapted from Paxinos and Franklin, 2001) showing the 28 brain nuclei where c-Fos-ir cells were quantified. Number below each diagram indicates the distance from Bregma. **(C)** Numbers of c-Fos-ir cells in 28 brain nuclei of difopein- [*n* = 9 (5 males and 4 females)] or YFP-injected [*n* = 7 (4 males and 3 females)] mice following OFT (Student’s unpaired two-tailed t-tests; Cg, *p = 0.0133; LSr, *p = 0.0102; LSc, **p = 0.0069; BST, *p = 0.0466; LH, *p = 0.0255; RT, *p = 0.0115; dCA1, ***p < 0.0001; dCA3, *p = 0.0286; DG, *p = 0.0189; Sub, ***p = 0.0003). **(D)** Left, schematic illustration of dual anterograde and retrograde tracing strategy. Right, representative confocal images of CTB-labeled VTA-projecting cells (green) and dHP efferent projections (red) overlapping the LS. Scale bar = 200 μm (100 μm for zoom in image). **(E,F)** Representative confocal images of VTA-projecting CTB-labeled cells in the LS colocalized with GAD67-ir **(E)** or GABA-ir cells **(F)**. Scale bar = 10 μm. **(G)** Left, schematic illustration of retrograde tracer CTB488 unilaterally injected into the LS. Right, representative confocal image of CTB-labeled LS-projecting cells in the dCA1. Scale bar = 200 μm. All data are presented as mean ± SEM. Data points from male and female mice are shown as black and red symbols, respectively.

As the dCA1 does not directly project to the VTA, we sought to identify the intermediary brain nuclei through which 14-3-3 dysfunction in the dCA1 indirectly modulates VTA activity during OFT. Previous work has established that OFT induces c-Fos expression in several brain regions (Hale et al., 2008; Bourgeois et al., 2012). As the difopein expression in the dCA1 induces locomotor hyperactivity in WT mice, the resulting c-Fos level alterations in specific brain nuclei likely reflect their involvement in the dCA1-VTA pathway. Therefore, we assessed the differences in c-Fos expression between difopein- and YFP-injected mice in response to OFT. Specifically, we examined 28 brain regions previously reported to be associated with either OFT or DA-associated hyperlocomotive behaviors (Badiani et al., 1998; Hale et al., 2008; Strange et al., 2014; Beier et al., 2015; Figures 4A,B). Interestingly, we found that AAV-mediated difopein expression in the dCA1 results in robust c-Fos expression in the dCA1 (Figure 4C and Supplementary Figure 1), suggesting that 14-3-3 dysfunction may lead to over-excitation of the dCA1 neurons during OFT. Of note, significantly higher numbers of c-Fos-ir cells were also identified in the cingulate cortex (Cg), LS, bed nucleus of the stria terminalis (BST), lateral hypothalamus (LH), reticular thalamic nucleus (RT), and other subregions of the hippocampus in difopein-injected mice compared to control (Figure 4C and Supplementary Figure 2). Among these identified brain regions, difopein-expressing dCA1 efferent projections were particularly evident in the medial part of the caudal LS (LSc) and the dorsomedial part of the rostral LS (LSr; Supplementary Figure 2).

The LS is predominantly composed of GABAergic neurons and is involved in motivated behavior, addiction, anxiety, and affect by integrating multiple sensory inputs and adjusting behaviors in response to environmental stimuli (Sheehan et al., 2004; Luo et al., 2011; Jiang et al., 2018; Leroy et al., 2018). Additionally, several lines of evidence support the involvement of the LS in psychiatric disorders, while its precise role remains unclear (Sheehan et al., 2004). To verify the role of the LS in connecting the dCA1 and the VTA, we unilaterally injected an anterograde virus (AAV-CaMKIIa-tdTomato) into the dCA1 and a retrograde tracer (CTB488) into the ipsilateral VTA of WT mice (Figure 4D). We found that tdTomato-filled dCA1 axons innervate the medial parts of the LSc and the LSr (Figure 4D), which is identical to the projection patterns observed in the LS of difopein-injected mice (Supplementary Figure 2). CTB-labeled VTA-projecting cells were found in both the LSc and LSr, including the regions where dHP axons innervate (Figure 4D). Using immunohistochemistry against GAD67 or GABA, these VTA-projecting LS cells were identified to be GABAergic (Figures 4E,F). Finally, we injected CTB488 into the LS and observed retrogradely labeled cells in the dHP CA1, suggesting that the dCA1 makes monosynaptic connection with the LS (Figure 4G). Together, these results demonstrate that the LS is over-activated during difopein-induced locomotor hyperactivity and anatomically connected with both the dCA1 and the VTA.

### 14-3-3 Inhibition in dCA1 Increases the Number of C-Fos-Expressing VTA-Projecting LS Neurons Following Open Field Exposure

**Figure 5:**
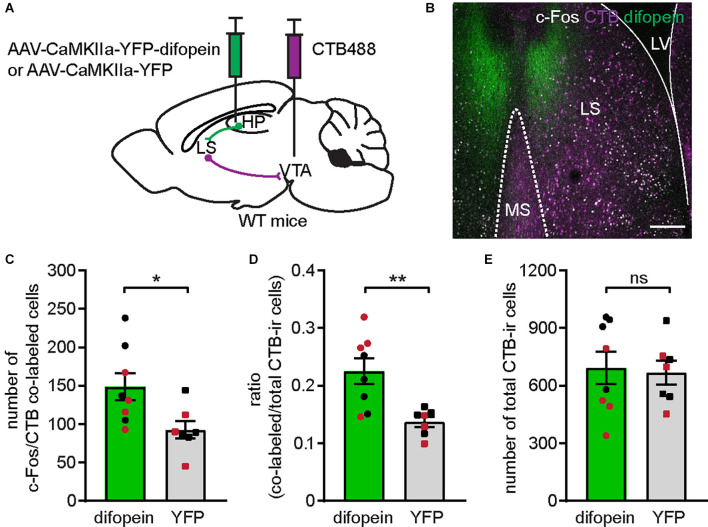
Inhibition of 14-3-3 in the dCA1 activates VTA-projecting LS cells. **(A)** Schematic illustration of AAV-CaMKIIa-YFP-difopein (or AAV-CaMKIIa-YFP) and CTB488 bilaterally injected into the dHP CA1 and VTA of WT mice, respectively. **(B)** Representative confocal image of c-Fos-ir cells (white) and VTA-projecting CTB-ir cells (purple) in the LS of a difopein-injected mouse. Scale bar = 200 μm. **(C–E)** Comparison of the numbers of c-Fos/CTB co-labeled cells **(C)** (Student’s unpaired two-tailed t-test, *p = 0.0237); the ratio of c-Fos/CTB co-labeled cells to total CTB-ir cells **(D)** (Student’s unpaired two-tailed t-test with Welch’s correction, **p = 0.0049); and the total number of CTB-ir cells **(E)** (Student’s unpaired two-tailed t-test, *p* = 0.8270) in the LS of difopein-CTB [*n* = 8 (4 males and 4 females)] or YFP-CTB [*n* = 7 (4 males and 3 female)] injected mice following OFT, indicating an elevated LS-VTA projection associated with 14-3-3 inhibition in the dHP during OFT. All data are presented as mean ± SEM. Data points from male and female mice are shown as black and red symbols, respectively. ns, not significant.

Having observed that difopein expression in the dCA1 leads to increased numbers of c-Fos-ir cells in the LS during locomotor hyperactivity, we next sought to determine whether the activities of the VTA-projecting LS neurons are altered as a result of 14-3-3 inhibition in the dCA1. In this experiment, AAV-difopein (or AAV-YFP as control) and CTB488 were bilaterally injected into the dCA1 and VTA of WT mice, respectively (Figures 5A,B and Supplementary Figure 3). In response to OFT, a significantly higher number of c-Fos-ir/CTB-ir co-labeled cells were found in the LS of difopein-injected mice compared to control (Figures 5C–E), indicating an increased activation of VTA-projecting LS neurons. As the LS is populated by GABAergic neurons (Figures 4E,F; Onténiente et al., 1987; Risold and Swanson, 1997), this result suggests that an increased activation of inhibitory projection from the LS to the VTA is associated with difopein-induced locomotor hyperactivity.

### Chemogenetic Inhibition of the LS Attenuates Difopein-Induced Locomotor Hyperactivity and DA Neuron Over-Activation

**Figure 6:**
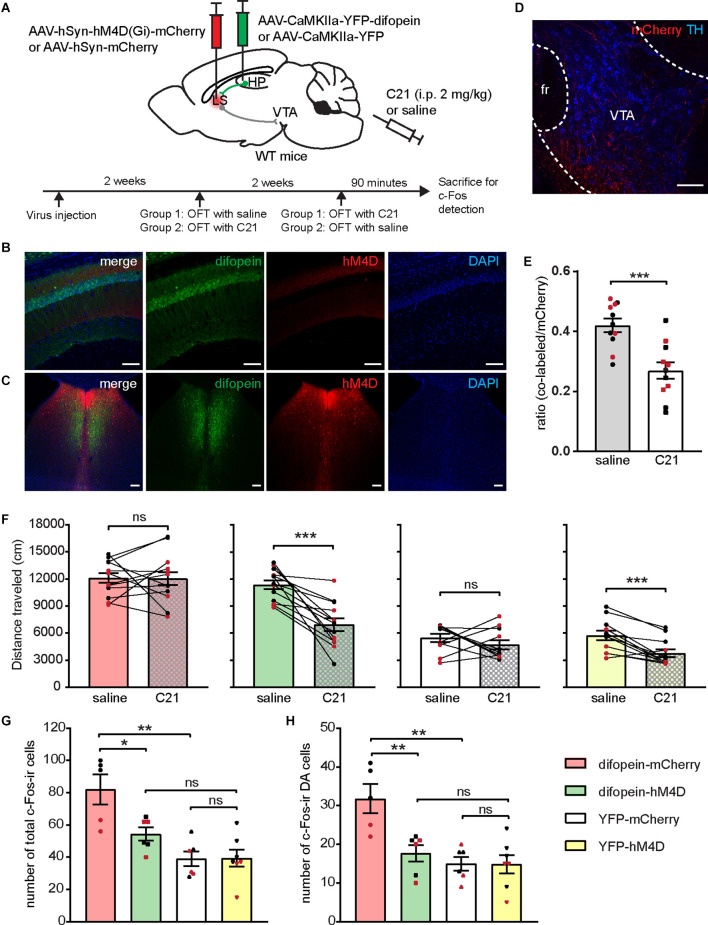
Chemogenetic inhibition of the LS attenuates 14-3-3 inhibition induced locomotor hyperactivity and DA neuron over-activation. **(A)** Schematic illustration of viral transductions and experimental timeline. **(B–D)** Representative images of difopein and hM4D expression in the dCA1 **(B)** and the LS **(C)**, as well as efferent projections from mCherry-infected LS neurons in the VTA **(D)**. Scale bar = 100 μm. **(E)** Ratio of c-Fos/hM4D co-labeled cells to total hM4D-expressing cells in the LS during OFT following saline or C21 treatment (*n* = 11 sections from 2 male and 2 female mice/group, Student’s unpaired two-tailed t-test, ***p = 0.0004), indicating an effective chemogenetic inhibition of LS neural activity. **(F)** Distance traveled of injected mice in OFT following either saline or C21 administration. Student’s paired two-tailed t-test: difopein-mCherry [*n* = 12 (5 males and 7 females), *p* = 0.9711]; difopein-hM4D (*n* = 13 (7 males and 6 females), ***p = 0.0001); YFP-mCherry [*n* = 11 (6 males and 5 females), *p* = 0.3457]; and YFP-hM4D [*n* = 12 (6 males and 6 females), ***p = 0.0004]. **(G,H)** Number of total c-Fos-ir **(G)** and c-Fos-ir DA cells **(H)** in the VTA of injected mice during OFT following C21 administration: difopein-mCherry (*n* = 3 males and 2 females), difopein-hM4D (*n* = 3 males and 3 females), YFP-mCherry (*n* = 3 males and 3 females), and YFP-hM4D (*n* = 4 males and 3 females). Student’s unpaired two-tailed t-tests. **(G)** difopein-mCherry vs. difopein-hM4D, *p = 0.0176; difopein-mCherry vs. YFP-mCherry, **p = 0.0017; difopein-hM4D vs. YFP-hM4D, *p* = 0.0544; YFP-mCherry vs. YFP-hM4D, *p* = 0.9532. **(H)** difopein-mCherry vs. difopein-hM4D, **p = 0.0075; difopein-mCherry vs. YFP-mCherry, **p = 0.0019; difopein-hM4D vs. YFP-hM4D, *p* = 0.4051; YFP-mCherry vs. YFP-hM4D, *p* = 0.9634. All data are presented as mean ± SEM. Data points from male and female mice are shown as black and red symbols, respectively. ns, not significant.

We then asked whether activation of the LS is necessary for difopein-induce locomotor hyperactivity. To address this question, we bilaterally injected AAV-hSyn-hM4D(Gi)-mCherry (or AAV-hSyn-mCherry as control) into the LS of WT mice in addition to AAV-difopein (or AAV-YFP) into the dCA1 (Figures 6A–D and Supplementary Figure 4). Administration of C21 (2 mg/kg) significantly lowered the ratio of c-Fos-ir hM4D(Gi)-expressing neurons to total hM4D(Gi)-expressing cells in the LS (Figure 6E and Supplementary Figure 4), indicating an effective suppression of LS neuronal activity. When assessed with an open field assay, we found that LS inhibition is sufficient to attenuate locomotor hyperactivity (Figure 6F). These results suggest that LS activation is necessary for locomotor hyperactivity induced by 14-3-3 inhibition in the dCA1.

To determine whether LS inhibition attenuates difopein-induced locomotor hyperactivity by modulating VTA DA neuronal activation, we analyzed the number of c-Fos-expressing cells in the VTA of C21 injected mice from each viral group following OFT. C21-induced LS inhibition significantly reduced the total number of c-Fos-ir cells in the VTA of difopein-hM4D mice compared to difopein-mCherry mice (Figure 6G and Supplementary Figure 5). Further cell-type-specific analysis showed that inhibition of the LS leads to a significantly lower number of c-Fos-ir DA neurons in difopein-hM4D mice compare with difopein-mCherry mice (Figure 6H). Collectively, these results indicate that LS activation is required for the increased activation of VTA DA neurons during locomotor hyperactivity induced by 14-3-3 inhibition in the dCA1.

### Chemogenetic Activation of dCA1 Imitates Difopein-Induced Behavioral and Neuronal Activity Alterations

**Figure 7:**
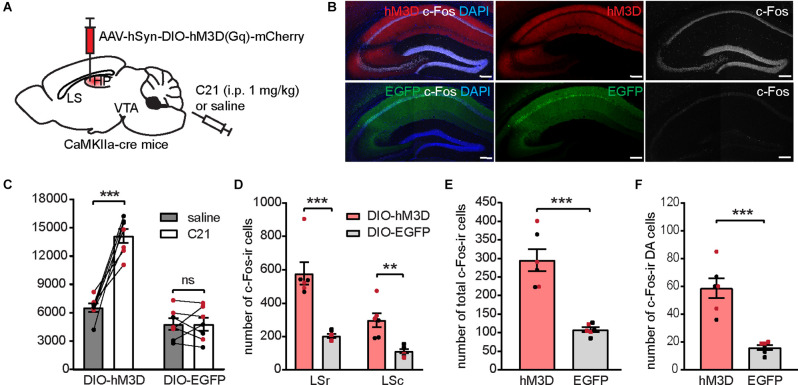
Chemogenetic activation of the dCA1 induces locomotor hyperactivity and increases c-Fos expression in the LS and the VTA. **(A)** Schematic illustration of AAV-hSyn-DIO-hM3D(Gq)-mCherry bilaterally transduced into the dHP CA1 of CaMKIIa-cre mice. **(B)** Representative confocal images showing hM3D activation by C21 effectively elicited c-Fos-ir (white) in the injection site. Scale bar = 200 μm. **(C)** Distance traveled of virally transduced CaMKIIa-cre mice during OFT following either saline or C21 administration [*n* = 7 (3 males and 4 females)/group; Student’s paired two-tailed t-tests; DIO-hM3D, ***p = 0.0003; DIO-EGFP, *p* = 0.9929]. **(D–F)** Number of total c-Fos-ir cells in the LS **(D)**, number of total c-Fos-ir cells in the VTA **(E)**, and number of c-Fos/TH co-labeled cells in the VTA **(F)** of virally transduced mice following C21 administration [*n* = 6 (3 males and 3 females)/group; **(D)**, multiple Student’s unpaired two-tailed t-tests; LSr, ***p < 0.0001; LSc, **p = 0.004; **(E)**, Student’s unpaired two-tailed t-tests, ***p = 0.0001; **(F)**, Student’s unpaired two-tailed t-tests, ***p = 0.0002]. All data are presented as mean ± SEM. Data points from male and female mice are shown as black and red symbols, respectively. ns, not significant.

Having observed a robust c-Fos expression in dCA1 neurons during difopein-induced hyperlocomotive behavior (Figure 4C and Supplementary Figure 1), we hypothesized that 14-3-3 inhibition leads to increased neuronal activation in difopein-expressing dCA1 cells, which results in locomotor hyperactivity *via* the dCA1-LS-VTA pathway. If so, direct chemogenetic activation of the dCA1 should be sufficient to elicit the behavioral and molecular alterations seen in the difopein-injected mice. To selectively activate pyramidal neurons in the dCA1, we bilaterally injected AAV-hSyn-DIO-hM3D(Gq)-mCherry (or AAV-CMV-DIO-EGFP as control) into the dCA1 of CaMKIIa-cre mice (Figure 7A). Compared with saline administration, C21 (1 mg/kg) injection sufficiently induced robust c-Fos expression in hM3D-infected dCA1 pyramidal neurons (Figure 7B). With this approach, we assessed the locomotive activity of injected mice under saline or C21 administration, as well as c-Fos expression in the LS and the VTA following OFT with C21. We found that activation of the dCA1 significantly increased the locomotive activity of CaMKIIa-cre mice during OFT (Figure 7C). Furthermore, dCA1 activation induced significantly higher numbers of c-Fos-ir cells in both the LS (Figure 7D) and the VTA (Figure 7E). Specifically, we found an increased number of c-Fos-ir DA neurons in the VTA due to dCA1 activation (Figure 7F). Collectively, these results demonstrate that direct activation of the dCA1 is sufficient to reproduce behavioral as well as neuronal activity alterations in the dCA1-LS-VTA pathway which have been observed in mice with 14-3-3 inhibition in the dCA1. This suggests that 14-3-3 dysfunction might lead to over-activation of the affected pyramidal neurons, which in turn disrupt the neuronal activity within the downstream pathway.

### Chemogenetic Activation of LS-Projecting dCA1 Neurons Induces Locomotor Hyperactivity and Increases Neuronal Activation in the LS and VTA

**Figure 8:**
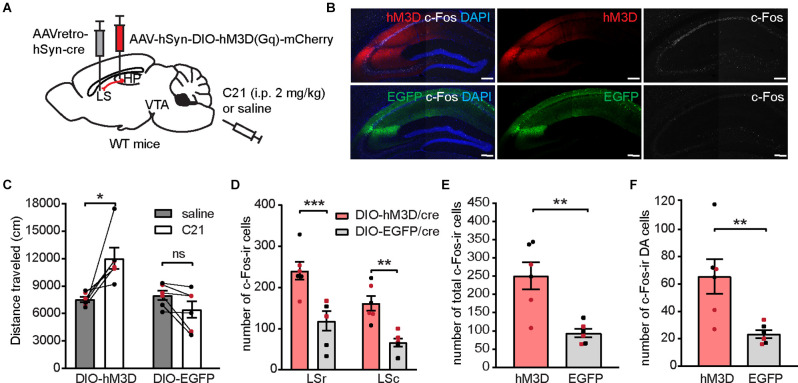
Chemogenetic activation of the LS-projecting dCA1 neurons induces locomotor hyperactivity and increases c-Fos expression in the LS and the VTA. **(A)** Schematic depiction illustrating viral transduction of AAV-hSyn-DIO-hM3D(Gq)-mCherry in the dHP CA1 and AAVretro-cre in the LS. **(B)** Representative confocal images showing hM3D activation by C21 effectively elicited c-Fos-ir (white) in LS-projecting CA1 neurons. Scale bar = 200 μm. **(C)** Distance traveled of virally transduced WT mice during OFT following either saline or C21 administration [*n* = 6 (3 males and 3 females)/group; Student’s paired two-tailed t-tests; DIO-hM3D/cre, *p = 0.0226; DIO-EGFP/cre, *p* = 0.6024]. **(D–F)** Number of total c-Fos-ir cells in the LS **(D)**, number of total c-Fos-ir cells in the VTA **(E)**, and number of c-Fos/TH co-labeled cells in the VTA **(F)** of virally transduced mice following C21 administration [*n* = 6 (3 males and 3 females)/group; **(D)**, multiple Student’s unpaired two-tailed t-tests; LSr, ***p < 0.0001; LSc, **p = 0.0019; **(E)**, Student’s unpaired two-tailed t-tests, **p = 0.0024; **(F)**, Student’s unpaired two-tailed t-tests, **p = 0.0090]. All data are presented as mean ± SEM. Data points from male and female mice are shown as black and red symbols, respectively. ns, not significant.

As dCA1 neurons project to several brain nuclei, we sought to determine whether activation of the dCA1-LS projection is sufficient to induce locomotor hyperactivity as well as neuronal activity alterations in the dCA1-LS-VTA pathway. To selectively activate dCA1-LS neurons, we bilaterally injected AAV-hSyn-DIO-hM3D(Gq)-mCherry (or AAV-CMV-DIO-EGFP as control) into the dCA1 of WT mice and a retrogradely propagating AAV encoding Cre-recombinase into the LS (Figures 8A,B). We found that activation of LS-projecting dCA1 neurons was sufficient to induce locomotor hyperactivity (Figure 8C), increased the number of c-Fos-ir cells in the LS (Figure 8D) and the VTA (Figure 8E), as well as increased the number of c-Fos-ir DA neurons in WT mice (Figure 8F). While the dCA1-LS pathway was specifically targeted to express hM3D, we found that LS-projecting dCA1 neurons also send collateral projections to the NAc and mammillary body (MM) to a lesser degree. As hippocampal bundles projecting to the NAc and MM pass through the LS, it is technically challenging to selectively activate dCA1 terminals in the LS without affecting the projections to other brain regions. Nonetheless, we investigated whether DREADD-mediated activation of NAc- or MM-projecting dCA1 neurons is sufficient to induce locomotor hyperactivity and found no significant changes in novelty-induced locomotor activity when either NAc- or MM-projecting dCA1 neurons were activated (Supplementary Figure 6). Considering that c-Fos expressions in either the NAc or MM were not different between difopein- and YFP-injected mice during OFT (Figure 4C), these results suggest that dCA1-LS projection is likely the primary pathway critical for psychomotor behavior induced by dCA1 hyperactivation.

**Figure 9:**
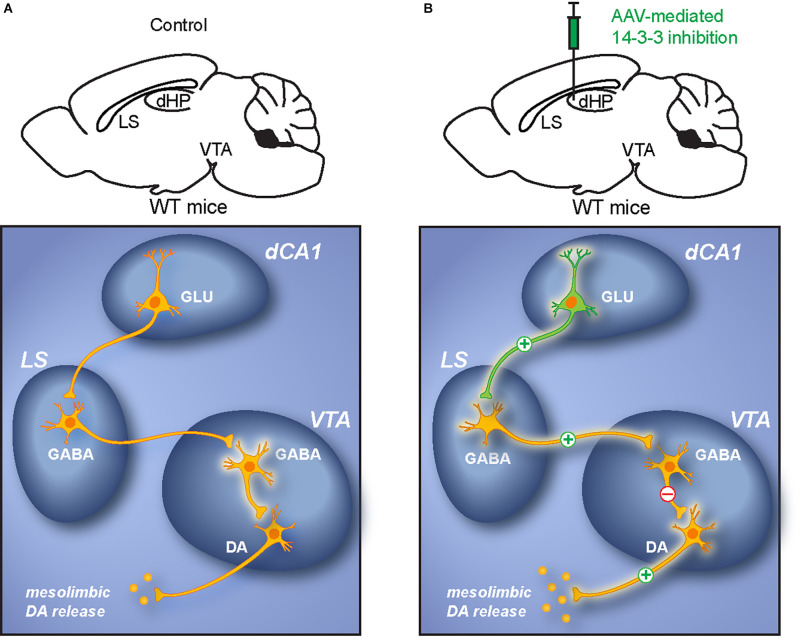
Proposed dCA1-LS-VTA pathway underlying psychomotor behavior. **(A)** Schematic illustration of the dCA1-LS-VTA polysynaptic pathway in WT mice. **(B)** Schematic illustration of alterations in neuronal activity within the pathway due to dCA1 dysfunction. The model proposes that 14-3-3 inhibition results in increased activation of pyramidal neurons in the dCA1, which leads to enhanced glutamatergic output to the LS that excites VTA-projecting GABAergic LS neurons. DA neurons in the VTA are then disinhibited by the elevated GABAergic projections from the LS. As a result, increased activation of VTA DA neurons induces psychomotor behaviors.

Together, the results of this study support a model in which 14-3-3 loss of function results in over-activation of the affected dCA1 pyramidal neurons. Such hippocampal dysfunction leads to increased activation of LS GABAergic neurons. Escalating inhibitory input from the LS to the VTA enhances DA neuronal activation *via* disinhibition (Vega-Quiroga et al., 2018), which ultimately induces psychomotor behavior (Figures 9A,B).

## Discussion

In this study, we focused on identifying the neural circuitry mechanism underlying 14-3-3 dysfunction in the dCA1 induced psychosis-like behavior in mice. Several lines of evidence from imaging studies of schizophrenia patients found decreased hippocampal volume and enhanced cerebral blood flow in both anterior and posterior HP (vHP and dHP in rodent; Medoff et al., 2001; Schobel et al., 2009a; Talati et al., 2014; McHugo et al., 2019). A loss of parvalbumin-containing GABAergic interneurons in the HP has also been found both in postmortem schizophrenia patients and in several animal models of schizophrenia (Zhang and Reynolds, 2002; Lodge et al., 2009; Marissal et al., 2018). Collectively, these findings implicate a robust hippocampal hyperactivity as an endophenotype in schizophrenia. As the HP detects novelty/familiarity by comparing previous memories with current sensory input, a dysfunctional HP may result in the aberrant assignment of salience that causes psychosis (Lisman and Grace, 2005; Kätzel et al., 2020; Modinos et al., 2020). While the precise relationship between hippocampal hyperactivity and mesolimbic DA dysregulation remains to be established in humans, studies using rodent models have provided some supporting evidence. For example, excessive neuronal firing in the ventral hippocampus (vHP) is found to be necessary for amphetamine-induced locomotor hyperactivity and increased DA neuron population activity in a prenatal methylazoxymethanol acetate rodent model of schizophrenia (Lodge and Grace, 2007). Optogenetic activation of vHP CA1/subiculum (vSub) neurons is sufficient to induce hyperlocomotion and cognitive deficits in WT mice (Wolff et al., 2018). Here, we demonstrated that the dorsal portion of the HP also plays an important role in the pathophysiology of psychosis-like behavior in mice, which is supported by previous studies: (1) serotonergic lesion of the dHP, but not the vHP, normalizes psychostimulant-induced locomotion and sensorimotor gating (Kusljic and van den Buuse, 2004; Adams et al., 2009); (2) c-Fos expression in the dHP is increased during psychomotor behavior in GluA1 knockout mice (Procaccini et al., 2011); and (3) chemogenetic activation of parvalbumin-containing interneurons in the dCA1 is sufficient to restore WT-like physiology and behaviors in Lgdel/+ mouse model of schizophrenia (Marissal et al., 2018). To delineate the neural circuitries underlying hippocampal regulation of DA activities, elegant studies were carried out illustrating a vSub/vCA1-nucleus accumbens-ventral pallidum-VTA pathway through which the vHP positively regulates VTA DA neuronal activities (Floresco et al., 2001; Lisman and Grace, 2005; Lodge and Grace, 2007, 2008, 2011). However, as dorsal and ventral portions of the HP have different anatomical connectivity and participate in distinct brain functions (Fanselow and Dong, 2010), it is possible that the dHP regulates the mesolimbic system *via* a distinct pathway. Furthermore, CA1 subfield abnormalities are especially highlighted in several human schizophrenia studies (Schobel et al., 2009b; Zierhut et al., 2013; Talati et al., 2014), yet the neural circuitry underlying dCA1’s regulation of DAergic activity in psychotic behavior has not been previously established. While we do not rule out the involvement of other interconnecting brain nuclei such as NAc, our results highlight a dCA1-LS-VTA polysynaptic pathway through which molecular disturbance of an important family of proteins in the dCA1 leads to a shift in E/I regulation of DAergic activity triggering psychosis-like behavior.

Here, we found that regional 14-3-3 inhibition enhances c-Fos expression in the dCA1 during OFT (Supplementary Figure 1), indicating that the affected dCA1 neurons are abnormally activated during novel environmental exposure. Additionally, we provided, to our knowledge, the first evidence that chemogenetic activation of LS-projecting dCA1 pyramidal neurons is sufficient to induce locomotor hyperactivity *via* the dCA1-LS-VTA pathway. This indicates that 14-3-3 inhibition-induced disturbance in downstream neuronal activities may be due to an increased dCA1 activation. This is particularly interesting as 14-3-3 deficiency has also been implicated in schizophrenia. Linkage analysis reveals that Ywhah, which encodes 14-3-3η, is located within the established 22q12-13 candidate risk chromosomal region of schizophrenia (Toyooka et al., 1999), though it is by no means the only 14-3-3 isoform linked with this disorder. Genetic and post-mortem mRNA analyses have identified decreased expression of other 14-3-3 isoforms in multiple brain regions of schizophrenia patients (Middleton et al., 2005; Wong et al., 2005; Ikeda et al., 2008; Kido et al., 2014). Within the past two decades, several animal models of schizophrenia with 14-3-3 deficiency were established (Cheah et al., 2012; Foote et al., 2015) and provided valuable insight into the functions of 14-3-3 at postsynaptic site and how 14-3-3 deficiency affects synaptic activities. It was shown that 14-3-3 deficiency is associated with a decrease in levels of NMDA receptor subunits, a significant reduction of the NMDA receptor-mediated synaptic currents in CA1 pyramidal neurons which express the 14-3-3 inhibitor, as well as an impairment of long-term potentiation at hippocampal CA3-CA1 synapses (Qiao et al., 2014; Foote et al., 2015; Graham et al., 2019). Additionally, one study shows that 14-3-3 binding slows desensitization kinetics of GluK2a-containing kainate receptor which mediates postsynaptic transmission, synaptic plasticity, and neuronal excitability (Sun et al., 2013). Moreover, 14-3-3 plays a significant role in synaptogenesis as 14-3-3 deficiency leads to a significant loss of the dendritic spine (Foote et al., 2015; Xu et al., 2015). However, it remains unclear what might be the molecular target of 14-3-3 that mediates dCA1 over-activation under 14-3-3 dysfunction. While we cannot determine solely with c-Fos expression whether 14-3-3 dysfunction-induced dCA1 activation is due to alterations in neuronal excitability, firing pattern, or receptor function, future studies using electrophysiology should help with future investigations of the precise mechanism. Nonetheless, given the critical roles 14-3-3 proteins play in synaptic transmission and plasticity, particularly in schizophrenia (Skoulakis and Davis, 1996; Broadie et al., 1997; Beguin et al., 2006; Li et al., 2006; Qiao et al., 2014; Chung et al., 2015), findings from this study should inspire novel therapeutic approaches for psychosis targeting 14-3-3-mediated neuronal processes. The LS receives strong glutamatergic input from the HP and is known to drive context-induced reinstatement *via* a dCA3-LS-VTA circuit (Luo et al., 2011), promote social aggression depending on dCA2 output (Leroy et al., 2018), and regulate feeding behaviors *via* vHP-LS pathways (Sweeney and Yang, 2015; Kosugi et al., 2021). Additionally, human studies have previously found abnormal septal structure and EEG activities in schizophrenia patients (Hanley et al., 1972; Heath and Walker, 1985), but the direct involvement of LS in psychosis-like behavior was previously undetermined. Here, we provide the following evidence that collectively supports a novel role of the LS in psychomotor behavior: (1) dCA1 dysfunction-induced locomotor hyperactivity and DA over-activation are associated with an over-activated LS as well as an elevated LS-VTA projection; (2) chemogenetic silencing of the LS is sufficient to attenuate both dCA1 dysfunction-induced locomotor hyperactivity and DA over-activation; (3) chemogenetic activation of dCA1-LS is sufficient to induce locomotor hyperactivity and DA over-activation. It is worth noting that while dCA1 projects predominantly to the dorsomedial part of the LS, other parts of the LS also contain c-Fos-ir cells induced by dCA1 dysfunction/activation. This might indicate the involvement of LS local interneurons that transmit hippocampal inputs within different subregions of the LS during locomotor hyperactivity, which will be investigated in future studies. Additionally, we showed that the LS positively regulates VTA DA neuronal activity, which is consistent with previous studies in which stimulation of the LS leads to activation of the DA neurons in the VTA by inhibiting local GABAergic activity (Luo et al., 2011; Vega-Quiroga et al., 2018). We presume that VTA GABAergic neurons play a similar role in the dCA1-LS-VTA pathway and will aim to elucidate such mechanisms in future studies.

In sum, our findings highlight a polysynaptic pathway through which 14-3-3 deficiency-induced dHP CA1 hyperactivation results in neural circuit abnormalities that lead to pathological DA dysregulation associated with psychomotor behavior. These results address a potential mechanism of psychosis and provide valuable insights and potential targets for future therapeutic development.

## Data Availability Statement

The original contributions presented in the study are included in the article/Supplementary Material, further inquiries can be directed to the corresponding author.

## Ethics Statement

The animal study was reviewed and approved by The Florida State University Animal Care and Use Committee.

## Author Contributions

JZ and YZ wrote the manuscript, conceived the project and designed the experiments. JZ performed all experiments, analyzed the data, and prepared the figures. YW assisted and supervised the maintenance of all mouse lines used in this study. MN assisted in genotyping and histology analysis. All authors contributed to the article and approved the submitted version.

## Conflict of Interest

The authors declare that the research was conducted in the absence of any commercial or financial relationships that could be construed as a potential conflict of interest.

## Publisher’s Note

All claims expressed in this article are solely those of the authors and do not necessarily represent those of their affiliated organizations, or those of the publisher, the editors and the reviewers. Any product that may be evaluated in this article, or claim that may be made by its manufacturer, is not guaranteed or endorsed by the publisher.
