# Supplementary material for: 14-3-3 Dysfunction in Dorsal Hippocampus CA1 (dCA1) Induces Psychomotor Behavior via a dCA1-Lateral Septum-Ventral Tegmental Area Pathway
Source: Front Mol Neurosci. 2022 Feb 14;15:817227. doi: 10.3389/fnmol.2022.817227 (PMC8882652; doi:10.3389/fnmol.2022.817227)
Supplement: Supplementary file 1 [file Data_Sheet_1.docx]

Supplementary Material

# Supplementary Figures


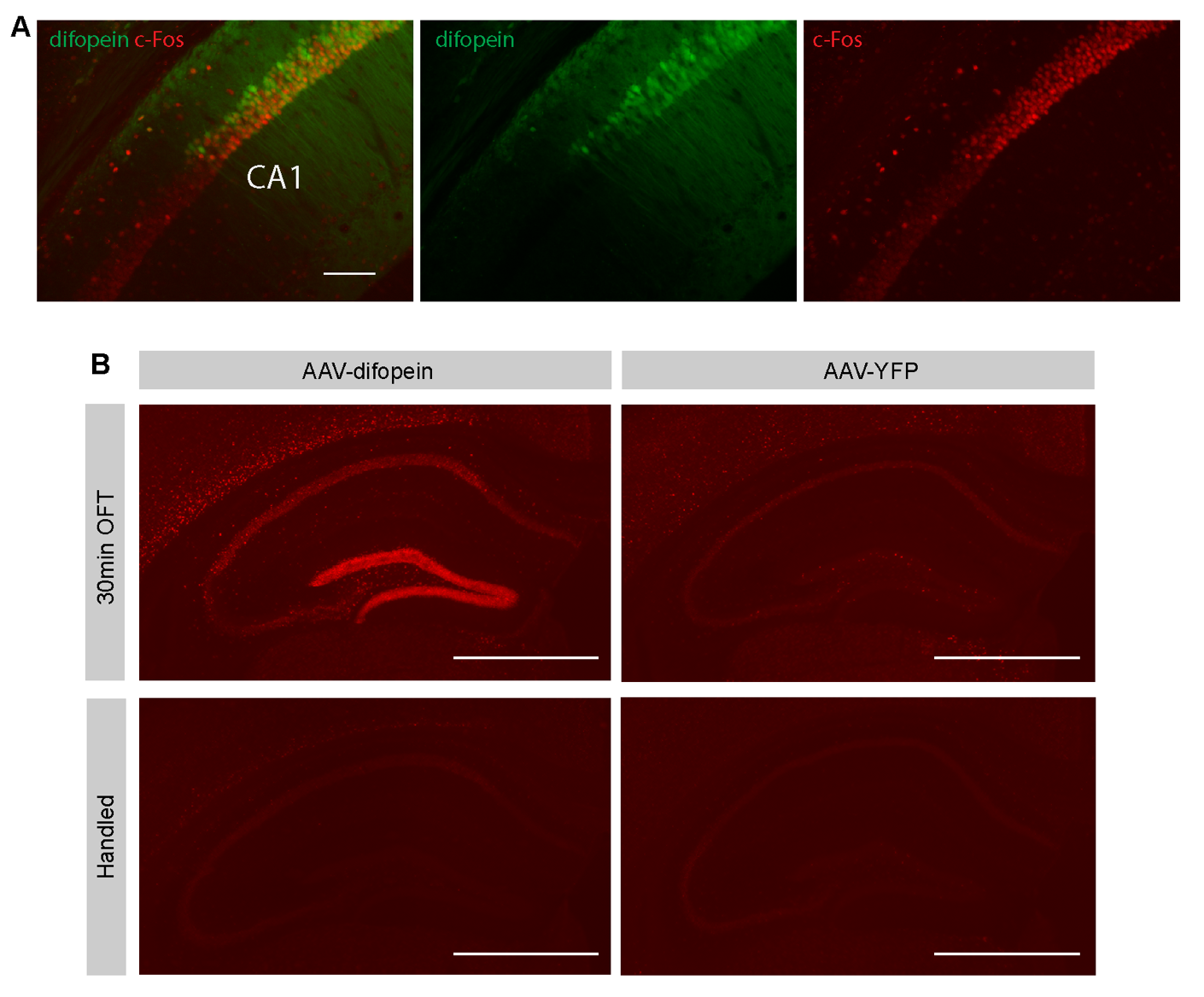


**Supplementary Figure 1.** AAV-mediated difopein expression results in robust c-Fos expression in the dHP CA1. **(A)** Representative image focusing on the edge of a difopein-infected CA1 area showing c-Fos-ir pyramidal cells were found in areas of the CA1 that were infected by AAV-CaMKIIa-YFP-difopein. Scale = 100 um. **(B)** Representative images showing c-Fos expression (red) patterns in the dCA1 of AAV-difopein or AAV-YFP injected mice following OFT or gentle handling. Scale = 1000 um.


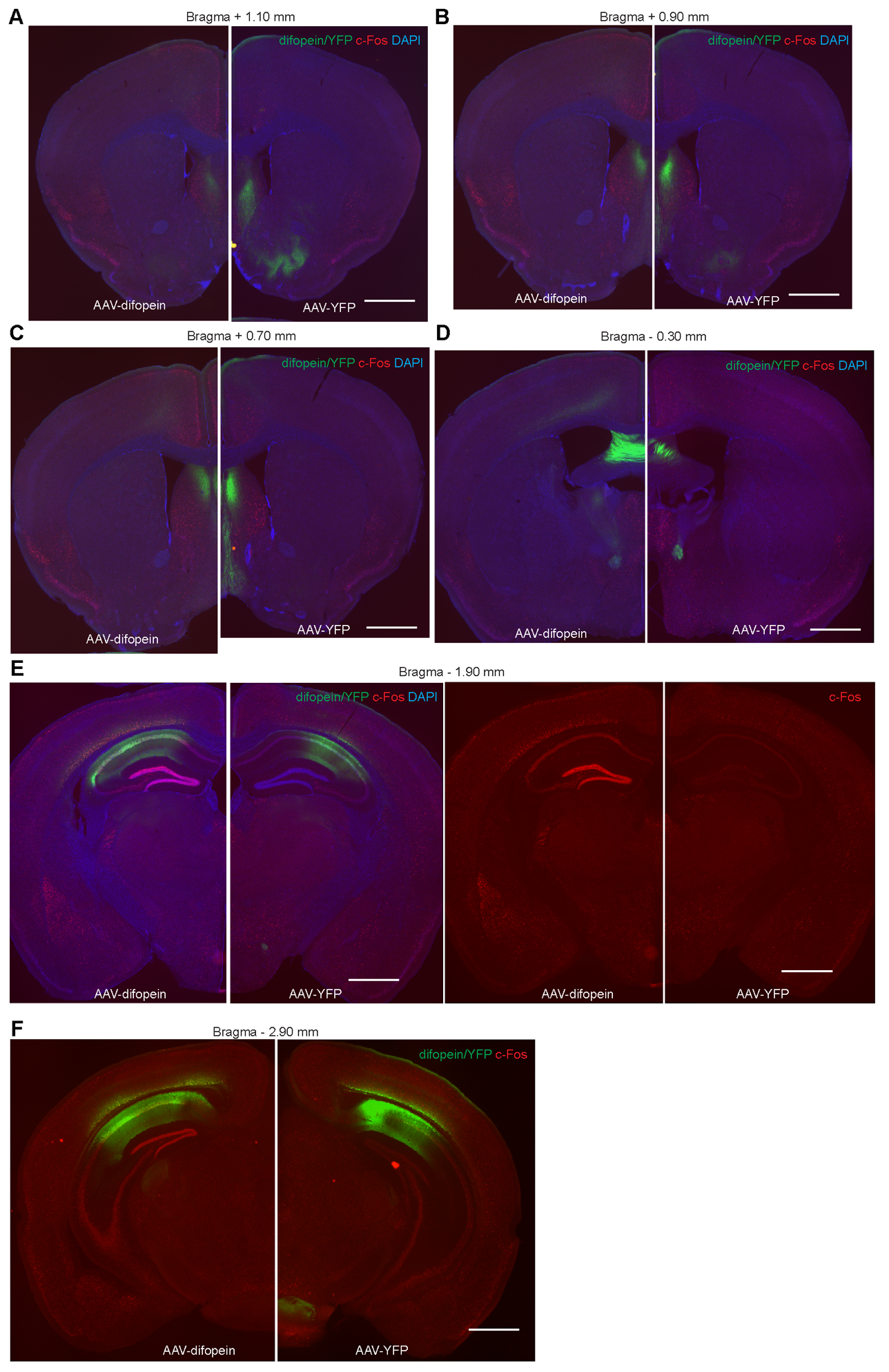


**Supplementary Figure 2.** Inhibition of 14-3-3 in the dCA1 results in c-Fos expression alterations in several brain nuclei. Representative images taken from six brain sections (**(A)** bregma + 1.10 mm, **(B)** bregma + 0.90 mm, **(C)** bregma + 0.70 mm, **(D)** bregma - 0.30 mm, **(E)** bregma - 1.90 mm, **(F)** bregma - 2.90 mm) comparing OFT-induced c-Fos expression (red) in several brain nuclei of difopein- or YFP-injected mice. Scale = 1 mm.


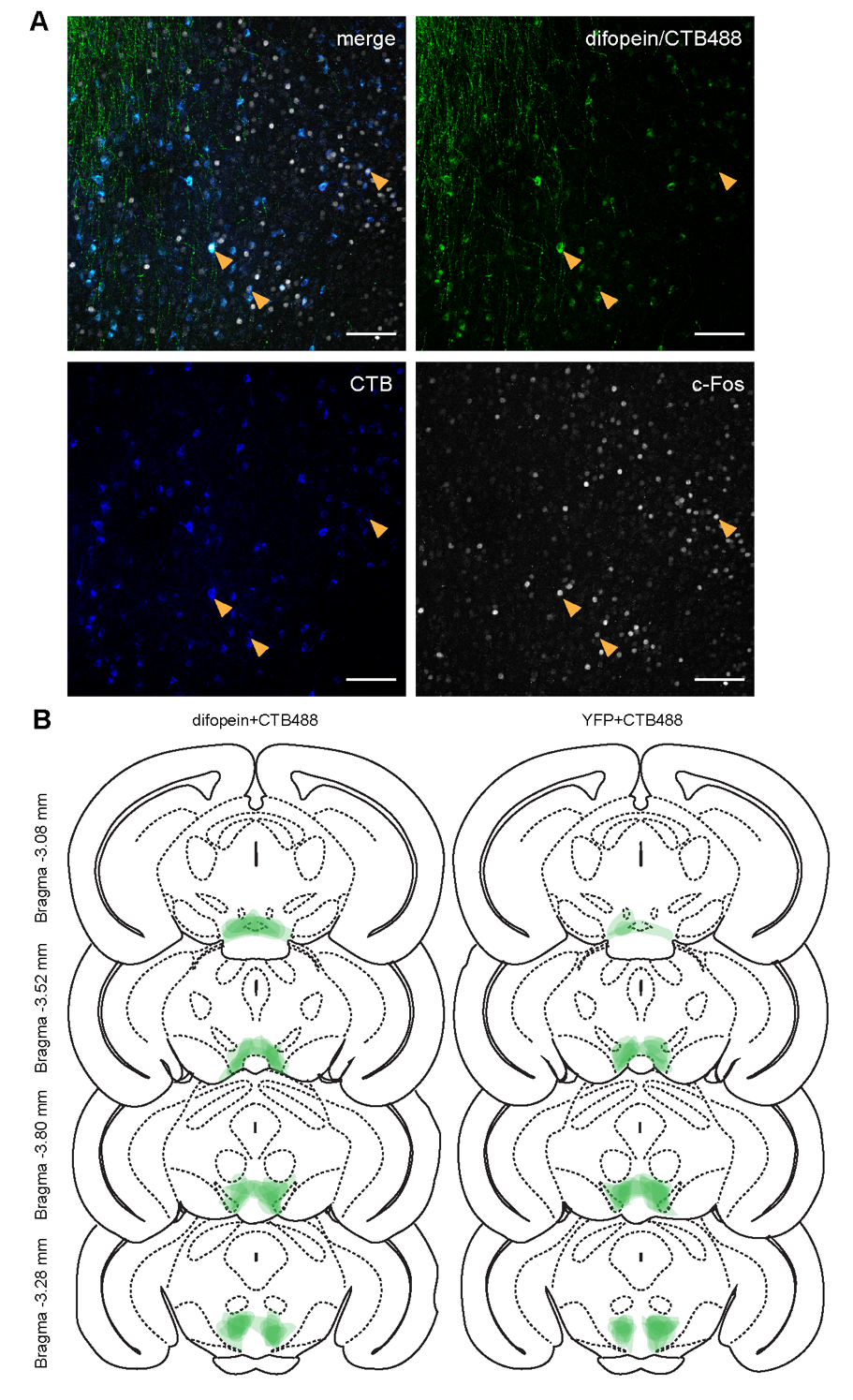


**Supplementary Figure 3.** Expression of c-Fos in VTA-projecting LS neurons. **(A)** Representative LS images from WT mice virally transduced with difopein in the dCA1 and CTB488 in the VTA. To separate CTB labeling from the difopein signal, immunohistochemistry against CTB was done using secondary antibody conjugated with DyLight™ 405. Orange arrowheads point to examples of c-Fos-ir cells that co-localized with CTB-ir cells. Scale = 100 um. **(B)** Schematic plots of CTB488 injection sites in difopein-CTB and YFP-CTB injected mice.


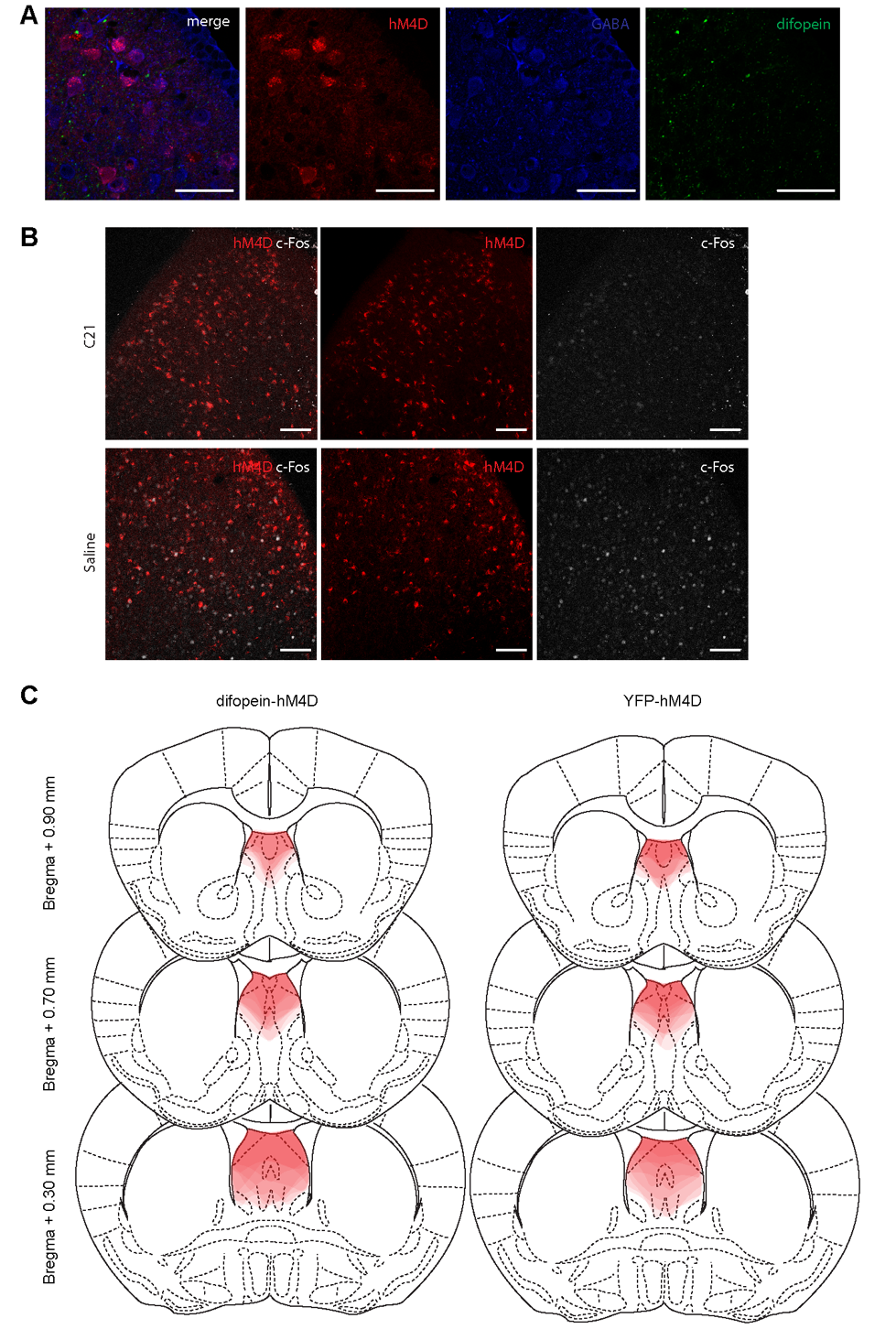


**Supplementary Figure 4.** Chemogenetic inhibition of the LS by expressing hM4D. **(A)** Representative images showing hM4D-expressing cells in the LS co-localized with GABA marker. Scale = 10 um. **(B)** Representative images showing OFT-induced c-Fos expression in the LS of difopein-hM4D mice following C21 (top) or saline (bottom) administration. Scale = 100 um. **(C)** Schematic plots of AAV-hSyn-hM4D(Gi)-mCherry injection sites in difopein-hM4D and YFP-hM4D mice.


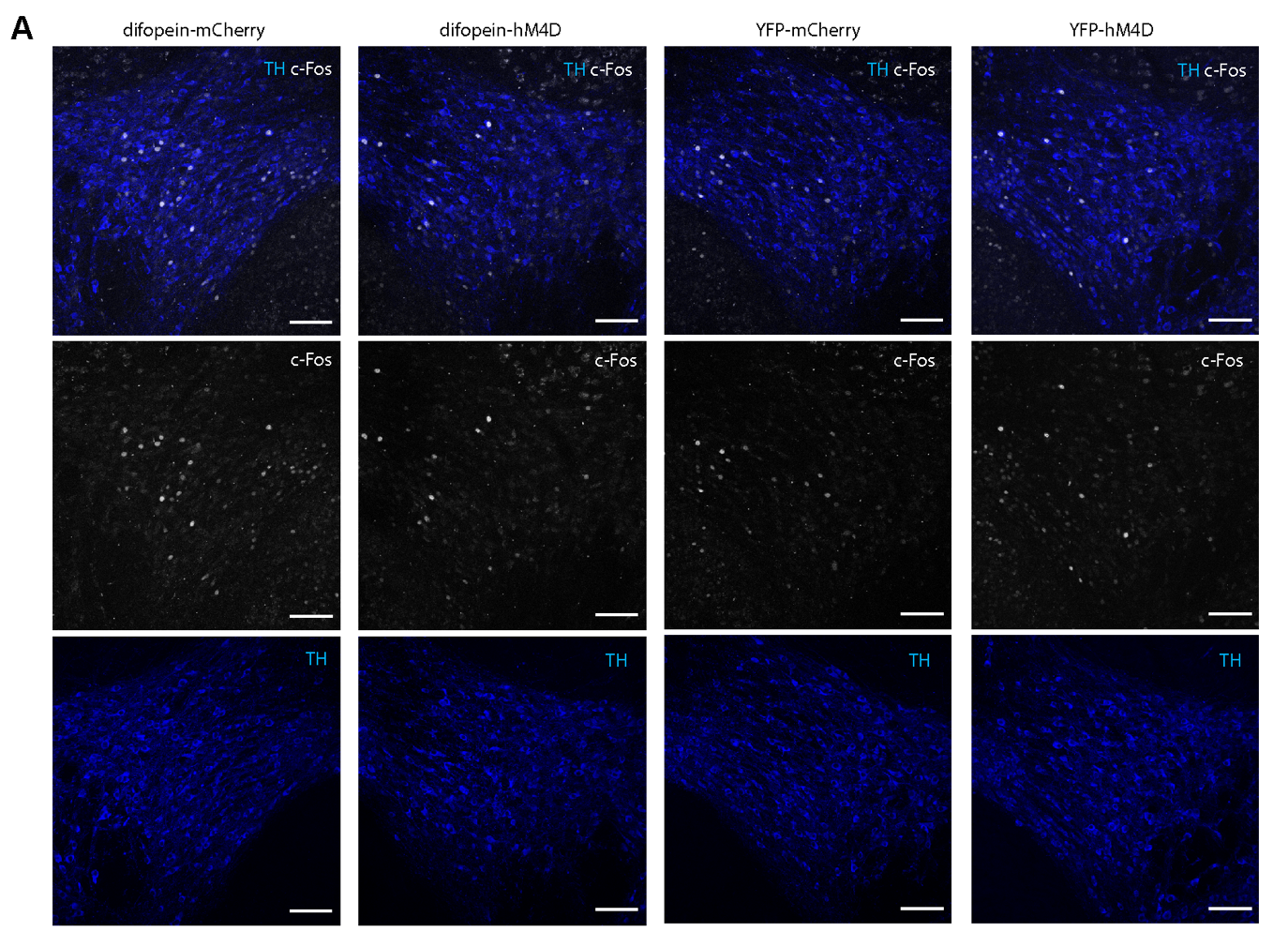


**Supplementary Figure 5.** Chemogenetic inhibition of the LS reduces c-Fos expression in the VTA of difopein-injected mice. **(A)** Representative images showing OFT-induced c-Fos expression in the VTA of difopein-mCherry, difopein-hM4D, YFP-mCherry, and YFP-hM4D mice following C21 administration. Scale = 100 um.


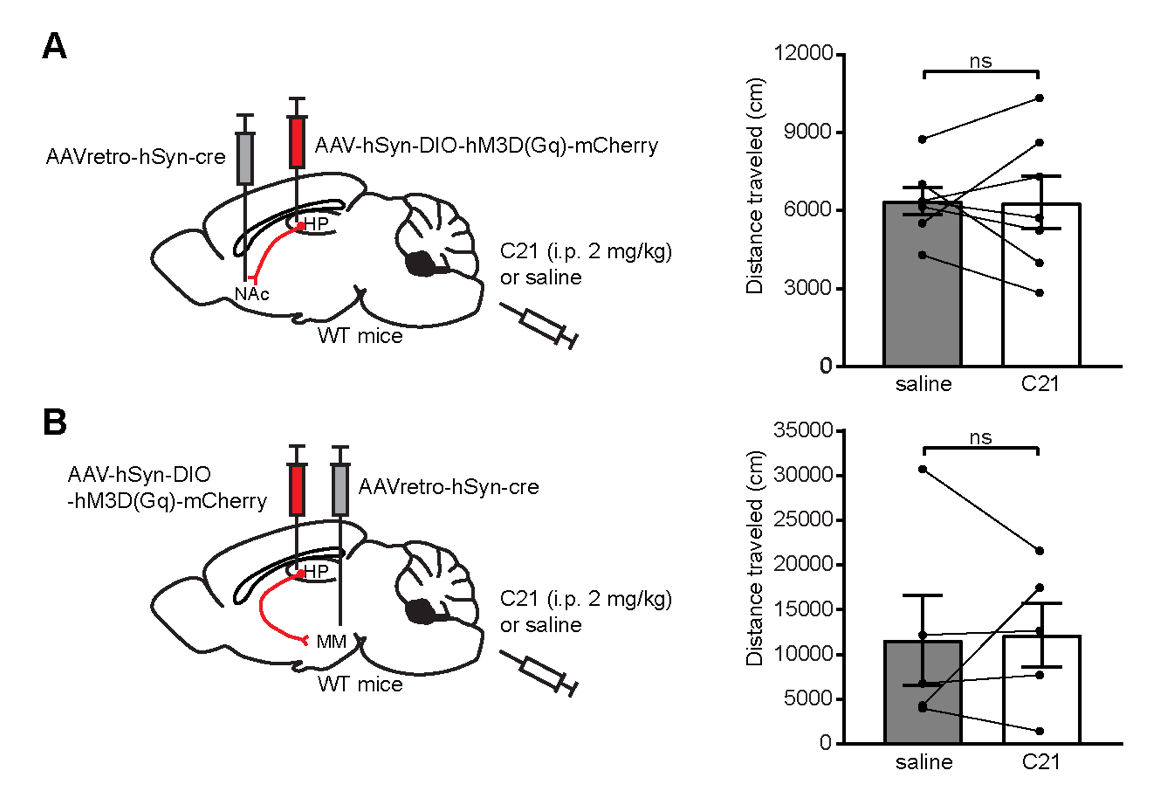


**Supplementary Figure 6.** Chemogenetic activation of NAc- or MM-projecting dCA1 neurons does not significantly increase locomotor activity of WT mice. **(A)** Left, schematic depiction illustrating viral transduction of AAV-hSyn-DIO-hM3D(Gq)-mCherry in the dCA1 and AAVretro-cre in the NAc. Right, distance traveled of virally transduced WT mice during OFT following either saline or C21 administration (n = 7, Student’s paired two-tailed t-tests, p = 0.9440). **(B)** Left, schematic depiction illustrating viral transduction of AAV-hSyn-DIO-hM3D(Gq)-mCherry in the dCA1 and AAVretro-cre in the MM. Right, distance traveled of virally transduced WT mice during OFT following either saline or C21 administration (n = 5, Student’s paired two-tailed t-tests, p = 0.8796). All data are presented as mean ± SEM.
